# Enzyme‐Empowered “Two Birds with One Stone” Strategy for Amplifying Tumor Apoptosis and Metabolic Clearance

**DOI:** 10.1002/advs.202308251

**Published:** 2024-03-06

**Authors:** Hanyue Li, Yihui Li, Lina Su, Ke Zheng, Yue Zhang, Jing Li, Feng Lv, Mengjie Huang, Ting Chen, Hanjie Zhang, Zhaoqing Shi, Dunwan Zhu, Xia Dong, Weiwei Zeng, Lin Mei

**Affiliations:** ^1^ State Key Laboratory of Advanced Medical Materials and Devices Tianjin Key Laboratory of Biomedical Materials Institute of Biomedical Engineering Chinese Academy of Medical Sciences and Peking Union Medical College Tianjin 300192 P. R. China; ^2^ Guangdong Provincial Key Laboratory of Malignant Tumor Epigenetics and Gene Regulation Guangdong‐Hong Kong Joint Laboratory for RNA Medicine Sun Yat‐Sen Memorial Hospital Sun Yat‐Sen University Guangzhou 510120 P. R. China; ^3^ Department of Pharmacy Qujing Medical College Qujing 655000 P. R. China; ^4^ School of Materials Science and Engineering Dongguan University of Technology Dongguan 523808 P. R. China; ^5^ Department of Nephrology First Medical Center of Chinese PLA General Hospital National Key Laboratory of Kidney Diseases National Clinical Research Center for Kidney Diseases Beijing Key Laboratory of Kidney Diseases Research Beijing 100853 P. R. China

**Keywords:** apoptosis amplification, enzymatic oxidation, metabolic clearance, rhenium nanoclusters, tumor defense

## Abstract

Nanomedicine has reshaped the landscape of cancer treatment. However, its efficacy is still hampered by innate tumor defense systems that rely on adenosine triphosphate (ATP) for fuel, including damage repair, apoptosis resistance, and immune evasion. Inspired by the naturally enzymatic reaction of glucose oxidase (GOx) with glucose, here a novel “two birds with one stone” technique for amplifying enzyme‐mediated tumor apoptosis and enzyme‐promoted metabolic clearance is proposed and achieved using GOx‐functionalized rhenium nanoclusters‐doped polypyrrole (Re@ReP‐G). Re@ReP‐G reduces ATP production while increasing H_2_O_2_ concentrations in the tumor microenvironment through GOx‐induced enzymatic oxidation, which in turn results in the downregulation of defense (HSP70 and HSP90) and anti‐apoptotic Bcl‐2 proteins, the upregulation of pro‐apoptotic Bax, and the release of cytochrome c. These processes are further facilitated by laser‐induced hyperthermia effect, ultimately leading to severe tumor apoptosis. As an enzymatic byproduct, H_2_O_2_ catalyzes the conversion of rhenium nanoclusters in Re@ReP‐G nanostructures into rhenate from the outside in, which accelerates their metabolic clearance in vivo. This Re@ReP‐G‐based “two birds with one stone” therapeutic strategy provides an effective tool for amplifying tumor apoptosis and safe metabolic mechanisms.

## Graphical abstract

A novel “two birds with one stone” therapeutic strategy based on GOx‐functionalized rhenium nanoclusters‐doped polypyrrole (Re@ReP‐G) inhibits ATP, defensive HSP, and anti‐apoptotic Bcl‐2 for hyperthermia‐enhanced enzyme‐mediated tumor apoptosis, while increasing H_2_O_2_ levels for enzyme‐promoted metabolic clearance.

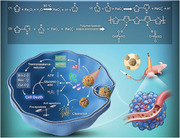

## 1 Introduction

The innate defense mechanisms of tumor cells, which include damage repair, anti‐apoptosis, and immune‐evasion mechanisms, are major causes of poor antitumor efficiency and susceptibility to tumor recurrence and metastasis.^[^ ^1^ ^]^ An appropriate supply of nutrients and energy is necessary to maintain cellular functions, particularly in injured tumor cells.^[^ ^2^ ^]^ Adenosine triphosphate (ATP), a crucial energy source in tumor cells that is produced predominantly by anaerobic glycolysis, is an essential component in driving these defense systems to protect and repair injured cells, in addition to ensuring tumor cell survival and proliferation.^[^ ^3^ ^]^ Thereinto, heat shock proteins (HSP), including HSP70 and HSP90, are ATP‐dependent molecular chaperones that are upregulated in response to external stresses to correct unfolded, misfolded, and/or denatured proteins, such as heat‐denatured proteins forming during classical photothermal therapy, resulting in the higher tumor cell thermoresistance.^[^ ^4^ ^]^ In addition, it has been demonstrated that highly expressed HSP impacts the expression of apoptosis‐related Bax/Bcl‐2 family members, the release of cytochrome c (Cyt‐C), and the activation of caspase‐3 by directly interacting with or indirectly modulating kinases, thereby preventing tumor cell apoptosis.^[^ ^5^ ^]^ Therefore, impairing ATP synthesis in tumor cells is critical for mitigating tumor defense capabilities and improving tumor cell vulnerability to apoptosis.

Glucose oxidase (GOx) is a natural oxido‐reductase in the body that catalyzes the oxidation of glucose to gluconic acid and hydrogen peroxide (H_2_O_2_) utilizing ambient molecular oxygen as an electron acceptor and has shown enormous potential in cancer starvation and Fenton therapies.^[^ ^6^ ^]^ GOx outperforms innate anaerobic glycolysis in consuming intracellular glucose, owing to its high catalytic efficiency and simplicity, leading to a substantial increase in H_2_O_2_ concentration while significantly reducing ATP production, which in turn results in energy fatigue, defense system depression, and cell injury.^[^ ^7^ ^]^ On this basis, a variety of elaborate nanocarriers, including hollow/porous nanomaterials, 2D nanomaterials, and polymers, have been exploited in recent decades to encapsulate and deliver GOx. Among them, Fenton nanomaterials, which not only serve as carriers that deliver GOx to tumor tissues but also catalyze the conversion of H_2_O_2_ (the enzymatic byproducts) into hydroxyl radicals that enhance the tumor‐killing effect, offer more artistical approaches.^[^ ^8^ ^]^ Nevertheless, little attention has been dedicated to nanocarrier biodegradability and biosafety, let alone any potential synergy with enzymatic reactions to accelerate metabolic clearance in vivo, both of which are equally important indicators, aside from to a strong therapeutic efficacy.

Polypyrrole (PPy) is synthesized through the one‐step oxidative polymerization of pyrrole monomers, oxidants, and stabilizers, and has been reported to be used as a nanocarrier for drug delivery,^[^ ^9^ ^]^ a photothermal agent to burn tumors,^[^ ^10^ ^]^ and a nanoenzyme to activate immune response,^[^ ^11^ ^]^ due to its flexible structure, modifiability, high optical absorption, and biocompatibility. While the pharmacologically active components of oxidants and stabilizers are flourishingly focused on and optimized, another vital component of anions that run through the bulk to the surface via hydrogen bonding and electrostatic interactions with the pyrrolyl nitrogen moiety are frequently overlooked, but are critical for structural stability and tightness, as well as degradation characteristics.^[^ ^12^ ^]^ The more doped the anions are, the easier and faster degradation will be, especially through the introduction of degradation blasting sites based on anion precursors, such as popular platinum quantum dots formed through the reduction of tetrachloroplatinic acid.^[^ ^13^ ^]^ In terms of biodegradation and metabolic pathways, non‐toxic ionic metabolites are more likely to be eliminated from the body and are therefore more likely to be translated to the clinic than small fragments. As a result, the development of biodegradable nanomaterials, particularly those containing ion‐based degradation products, is crucial for biological applications.

**Scheme 1:**
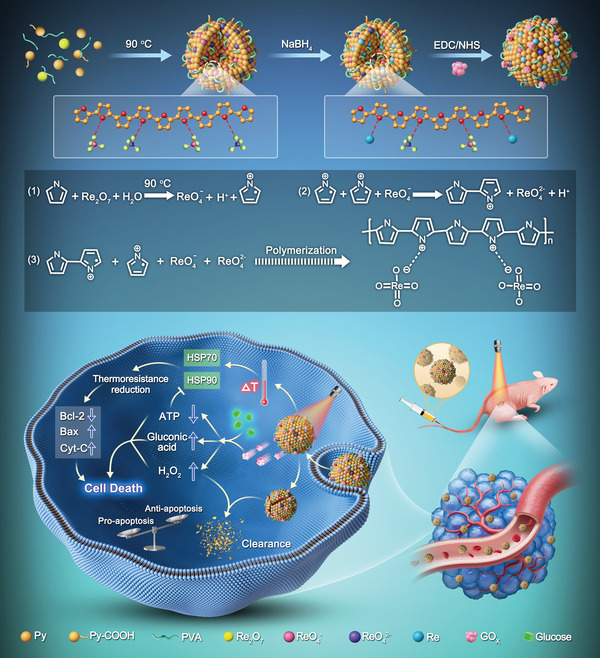
The schematic of synthetic procedure for Re@ReP‐G and the underlying mechanism of enzyme‐mediated apoptosis amplification and enzyme‐promoted metabolic clearance.

Herein, an intriguing rhenium (Re) nanoclusters‐doped PPy (termed as Re@ReP) was facilely synthesized through oxidative polymerization using the oxidant dirhenium heptaoxide (Re_2_O_7_) and subsequent in situ reduction of rhenic acid using the reductant sodium borohydride (NaBH_4_) (**Scheme** **1**). Following GOx modification via amide bonding, the corresponding product Re@ReP‐G catalyzed the oxidation of intratumoral glucose to gluconic acid and H_2_O_2_, which decreased the ATP yield and downregulated HSP70 and HSP90 expression, eventually leading to the attenuation of tumor defense and anti‐apoptotic capabilities. Moreover, the induced hyperthermia in the Re@ReP‐G+L group led to higher tumor apoptosis and regression when subjected to 1064 nm laser irradiation, disregarding relatively moderate thermoresistance. On the other hand, H_2_O_2_, as an enzymatic byproduct, reacted with Re nanoclusters that permeate the bulk and surface of Re@ReP‐G, thereby accelerating their biodegradation and metabolic excretion in vivo. In general, this innovative Re@ReP‐G utilizing enzyme‐empowered “two bird with one stone” strategy showed promising clinical translation in cancer therapy due to its definite body clearance, high effectiveness, and excellent biosafety.

## 2 Results and Discussion

### 2.1 Synthesis and Characterization

**Figure 1:**
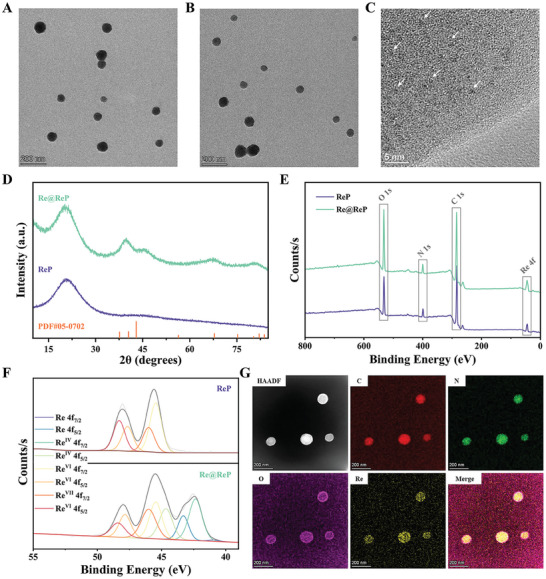
TEM images of A) ReP and B) Re@ReP. C) HRTEM images of Re@ReP. The white arrows indicate Re nanoclusters. D) XRD patterns, E) XPS spectra, and F) high‐resolution Re 4f XPS spectra of ReP and Re@ReP. G) Elemental mapping images of Re@ReP.

Taking advantage of the thermal hydrolysis of the oxidant Re_2_O_7_ to produce perrhenic acid (ReO_4_^−^), the pyrrole monomer was first oxidized to a radical cation by losing one electron, and then spontaneously dimerized with an adjacent radical cation to form a dihydromer dication with the concomitant transfer of one electron to ReO_4_^−^ to form rhenic acid (ReO_4_^2−^). In order to maintain electroneutrality, substantial amounts of ReO_4_^−^ and ReO_4_^2−^ were absorbed into the PPy skeleton during the polymerization process, resulting in the formation of rhenic acid‐doped PPy (termed as ReP) nanoparticles. According to transmission electron microscopy (TEM) images, the prepared ReP exhibited a spherical morphology with an average size of ≈82 nm (**Figure** **1**A). Following NaBH_4_ reduction, the resulting Re@ReP presented a similar morphology and hydrodynamic particle size (≈91 nm) to ReP, but with a lower zeta potential (−10.87 ± 0.25 mV) than ReP (21.23 ± 0.49 mV) (Figure 1B; Figure S1, Supporting Information). The polydispersity index of Re@ReP (0.181 ± 0.023) was slightly increased compared to ReP (0.134 ± 0.024). Through manipulation of specific component addition amounts (NaBH_4_, GOx, or pyrrole) while maintaining constant other parameters, we discovered that only pyrrole exhibited a correlation with the particle size and morphology of the final stabilized nanoparticles (Figure S2, Supporting Information). To identify the structural characteristics and chemical compositions of ReP and Re@ReP, high‐resolution TEM (HRTEM) images, X‐ray diffraction (XRD) spectroscopy, and X‐ray photoelectron spectroscopy (XPS) were carried out. As displayed in Figure 1C, ultrasmall Re nanoclusters were disseminated in Re@ReP nanostructures from the inside out, which visually demonstrated the successful formation of Re nanoclusters. From XRD detection, Re@ReP instead of ReP featured characteristic diffraction peaks that correspond to Re nanoclusters (PDF#05‐0702), indicating the successful deposition of Re nanoclusters during reduction (Figure 1D). In terms of XPS detection, both ReP and Re@ReP exhibited C 1s, N 1s, O 1s, and Re 4f peaks (Figure 1E). More importantly, nulvalent, tetravalent, hexavalent, and septivalent Re states were further demonstrated by the high‐resolution Re 4f XPS spectra of Re@ReP, which displayed a broader absorption range from 39.8 to 50.4 eV than that (43–50.4 eV) of ReP (Figure 1F). Elemental mapping images and energy dispersive spectrometry (EDS) maps confirmed the homogeneous distribution of the elements C, N, O, and Re within Re@ReP (Figure 1G; Figure S3, Supporting Information). Overall, these results demonstrated the successful fabrication of ReP and Re@ReP.

### 2.2 Hyperthermia‐Enhanced Enzymatic Activity and Enzyme‐Accelerated Degradability

**Figure 2:**
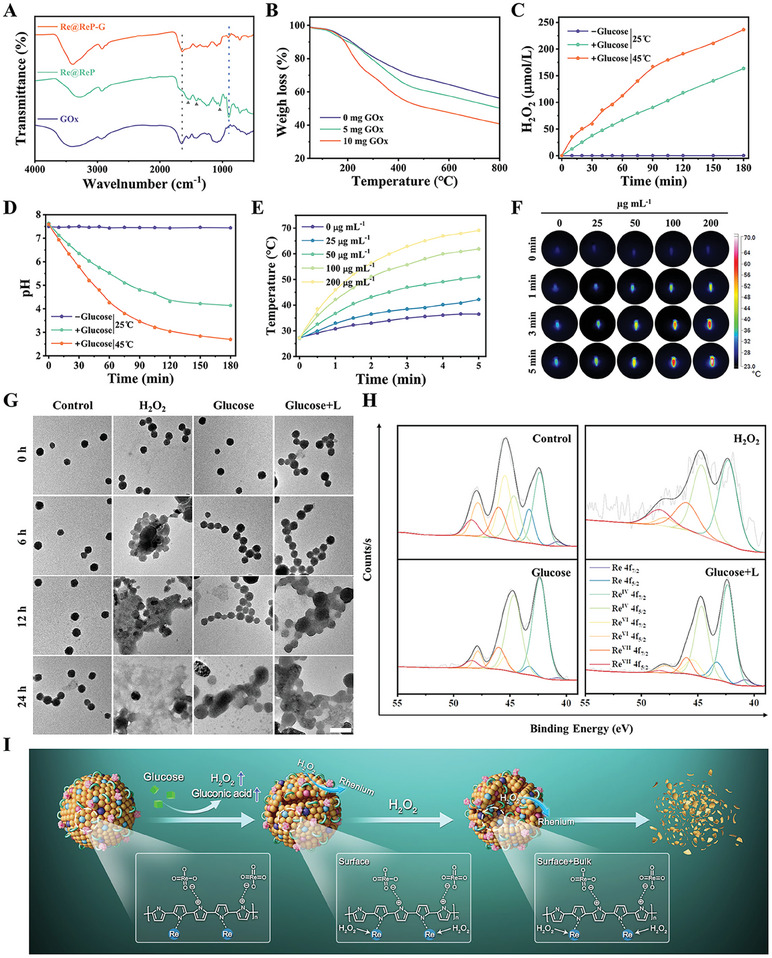
A) FTIR spectra of GOx, Re@ReP, and Re@ReP‐G. B) TGA curves of Re@ReP‐G with different adding amounts of GOx. C) H_2_O_2_ generation and D) pH decrease after different treatments. E) Temperature profiles of different concentrations of Re@ReP‐G under 1064 nm laser irradiation (1.0 W cm^−2^, 5 min) and F) the corresponding photothermal pictures. G) TEM images of Re@ReP‐G after different treatments for different time, scale bar: 200 nm. H) The high‐resolution Re 4f XPS spectrum of Re@ReP‐G after 24 h of different treatments. I) The underlying degradation mechanism of Re@ReP‐G.

Subsequently, GOx was covalently conjugated to the surface of Re@ReP through classic amidation reaction using NHS/EDC as coupling agents, which yielded the final product Re@ReP‐G. TEM images in Figure S4 (Supporting Information) revealed that GOx decoration had no apparent impact on the nanoparticles′ morphology or TEM size. As shown in Fourier transform infrared (FTIR) spectra in **Figure** **2**A, both Re@ReP and Re@ReP‐G emerged the characteristic peaks at 1046, 1420, and 1547 cm^−1^ belonging to the stretching vibrations of C─O, C─N, and C═C in the traditional PPy framework, respectively, suggesting the successful polymerization of pyrrole under stimulation of the Re_2_O_7_ oxidant. However, in contrast, the stretching vibration of Re═O and the amide bond of GOx were assigned to the absorption peaks at 906 and 1645 cm^−1^ in Re@ReP‐G, respectively, which provide further evidence for Re compound doping and GOx decoration. SDS‐PAGE protein analysis in Figure S5 (Supporting Information) also proved the successful conjugation of GOx. The thermogravimetric analysis (TGA) was used to determine the loading capacity of GOx, which was found to be 6.5% for 5 mg GOx addition and 15% for 10 mg GOx addition (Figure 2B). Moreover, Re@ReP‐G showed good stability and dispersibility over a period of 7 days in a variety of physiological media, including pure water, phosphate buffer saline (PBS), and Dulbecco's modified Eagle medium (DMEM) (Figure S6, Supporting Information).

To investigate the GOx catalytic activity, inherent photothermal property, and enzyme‐accelerated degradability of Re@ReP‐G, the enzyme activity assays, temperature monitoring, TEM images, and high‐resolution XPS spectra were performed. At first, the enzymatic activity was evaluated by incubating Re@ReP‐G in the presence or absence of glucose, which was reflected by the pH change (caused by gluconic acid) and H_2_O_2_ generation. Figure 2C,D illustrated that the presence of both glucose and Re@ReP‐G resulted in a steady drop in pH but an increase in H_2_O_2_ production over time and a certain increase in temperature favored enzymatic reactions of GOx. The employment of PPy‐based nanosystems as a photothermal agent for tumor ablation has been extensively studied in recent decades. Re@ReP‐G aqueous dispersion, as expected, exhibited broad and concentration‐dependent light absorption, as well as linearly improved absorbance with concentrations at wavelengths of 808 or 1064 nm, which laid the groundwork for effective photothermal conversion (Figure S7, Supporting Information). When exposed to an 808 or 1064 nm laser (1.0 W cm^−2^), Re@ReP‐G showed a rapid and evident dose‐dependent temperature increase, reaching as high as 69.1 and 61.9 °C, respectively, at relatively low concentration (100 µg mL^−1^) (Figures 2E,F and S8A,B, Supporting Information). Moreover, a laser power‐dependent temperature increase was also observed in Figure S8C (Supporting Information) and Figure S9A (Supporting Information). The similar heating and cooling temperature profiles of Re@ReP‐G after five on‐off cycles of laser irradiation proved their admirable photostability (Figures S8D and S9B, Supporting Information). Based on a single photothermal heating and cooling curve, the photothermal conversion efficiency of Re@ReP‐G was calculated to be 22.22% at 808 nm and 31.76% at 1064 nm (Figures S8E,F and S9C,D, Supporting Information). All of the foregoing results indicated that Re@ReP‐G was a potent photothermal agent capable of inducing photothermal therapy on malignancies.

In terms of enzyme‐accelerated degradability, Re@ReP‐G treated with H_2_O_2_ underwent distinct structural collapse and destruction at 12 h and further disintegration at 24 h, whereas the untreated group showed negligible changes in TEM morphology, which was attributed to the catalytic reaction of H_2_O_2_ with Re nanoclusters (Figure 2G). Due to the pace of enzymatic activity and the restricted synthesis of substrate H_2_O_2_, the Glucose+L group displayed gentler structural disruption than the H_2_O_2_ group but more serious decomposition behavior than the Glucose group without laser irradiation. This finding suggested that laser‐induced hyperthermia facilitated the enzymatic reaction of GOx and the generation of H_2_O_2_. To further analyze the final degradation form, high‐resolution XPS spectra were performed at the experimental endpoint. As shown in Figure 2H, the characteristic Re 4f_7/2_ and Re 4f_5/2_ peaks were less intense to varying degrees in the groups treated with Glucose and Glucose+L, but were completely absent in the spectra of the H_2_O_2_‐treated group, indicating that the final degradation form of Re@ReP‐G was primarily rhenate, which was theoretically easily metabolized and removed from the body. Based on those findings, an underlying mechanism for Re@ReP‐G degradation was proposed and illustrated in Figure 2I. When Re@ReP‐G reached tumor locations, intratumorally overexpressed H_2_O_2_ attacked Re nanoclusters on the surface first, while cargo GOx accelerated the oxidation of adjacent glucose to gluconic acid and H_2_O_2_. Higher quantities of H_2_O_2_ gained greater access to the inner Re nanoclusters and caused more severe damage from the inside out, resulting in drastic nanostructure destruction and ensuring good metabolism and biosafety.

### 2.3 In Vitro Cyto‐Dynamic Change and Tumor Apoptosis

**Figure 3:**
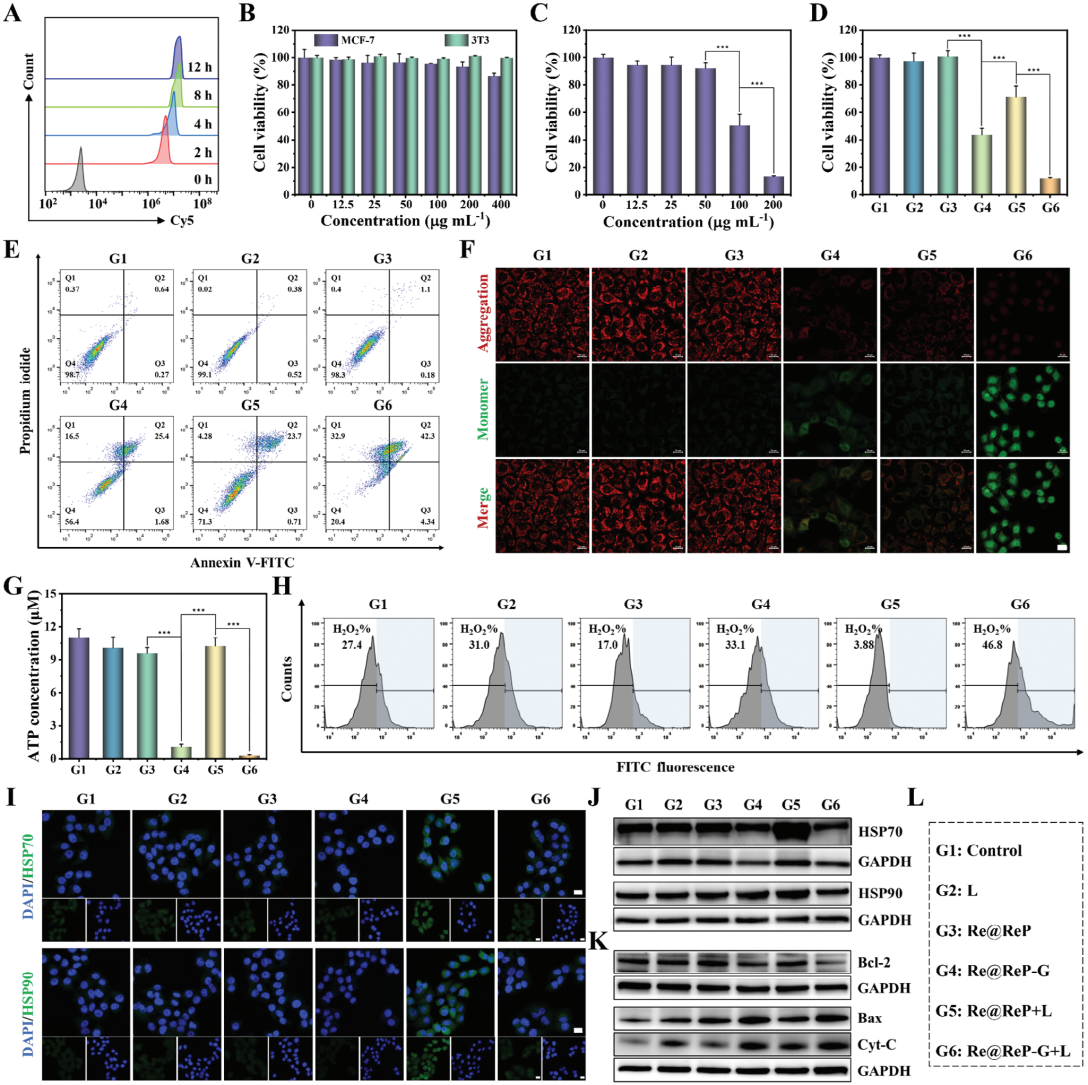
A) Flow cytometry of cellular internalization in MCF‐7 cells with Cy5‐labeled Re@ReP‐G for various times. B) Cell viability of MCF‐7 or 3T3 cells incubated with various doses of Re@ReP for 24 h. C) Cell viability of MCF‐7 cells incubated with various doses of Re@ReP‐G for 24 h. D) Cell viability of MCF‐7 cells after different treatments at equal Re@ReP concentration of 75 µg mL^−1^. E) Flow cytometry of cell apoptosis in diverse treatment groups. F) CLSM images of mitochondrial membrane potential after different treatments, scale bar: 20 µm. G) ATP and H) H_2_O_2_ levels in MCF‐7 cells treated with different formulations. I) CLSM images of HSP70 and HSP90 after different treatments, scale bar: 20 µm. J) Western blot assay of HSP70 and HSP90 expression. K) Western blot assay of Bcl‐2, Bax, and Cyt‐C expression. L) Different treatment groups. Statistical significance was calculated by one‐way ANOVA analysis. ^*^ *p* < 0.05; ^**^ *p* < 0.01; ^***^ *p* < 0.001.

Effective cellular internalization is a necessary condition for a positive therapeutic outcome, and was evaluated by labeling Re@ReP‐G with the Cy5 dye and subsequent detection using confocal laser scanning microscopy (CLSM) and flow cytometry. As shown in **Figures** **3**A and S10 (Supporting Information), Cy5‐labeled Re@ReP‐G was quickly phagocytosed by MCF‐7 cells in a time‐dependent manner, as confirmed by the progressively increasing Cy5 red fluorescence signal over time. Following that, the in vitro cytotoxicity of Re@ReP and Re@ReP‐G on MCF‐7 cancer cells or 3T3 normal cells was investigated using the standard cell counting kit‐8. No significant cytotoxicity to MCF‐7 and 3T3 cells was seen after 24 or 48 h of co‐incubation with any of the tested doses of Re@ReP, and >85% of the cells survived even after being exposed to the maximum dose of 400 µg mL^−1^ (Figure 3B; Figure S11, Supporting Information). The viability of MCF‐7 cells, however, rapidly declined with increasing Re@ReP‐G dose due to the GOx‐mediated enzymatic activity consuming glucose and ATP, and it further declined to 58.50% or 11.88% when treated with Re@ReP‐G+L at an equivalent Re@ReP dose of 50 or 75 µg mL^−1^, respectively (Figure 3C,D; Figure S12, Supporting Information). To further analyze the distribution of living and dead cells qualitatively and quantitatively, the specific probes calcein acetoxymethyl ester (Calcein‐AM)/Annexin V‐FITC and propidium iodide (PI) were applied, and the results revealed that Re@ReP‐G+L caused the most cellular damage in MCF‐7 cells, including 4.34% of early apoptosis, 42.3% of later apoptosis, and 32.9% of necrosis (Figure 3E; Figure S13, Supporting Information). Moreover, we adopted the JC‐1 kit to examine mitochondrial membrane alterations in MCF‐7 cells following treatment with Re@ReP‐G and other formulations. The green/red fluorescence intensity ratios were elevated in the Re@ReP‐G and Re@ReP+L groups, and further enhanced in the Re@ReP‐G+L group, suggesting mitochondrial membrane rupturing, which was ascribable to hyperthermia‐enhanced enzymatic reaction (Figure 3F). Collectively, Re@ReP‐G represented a distinct advantage in effective cellular internalization and remarkable tumor killing.

Mitochondrial membrane permeabilization is a signaling pathway that causes cell death, which can be counteracted by regulating the pro‐apoptotic Bax protein and the anti‐apoptotic Bcl‐2 protein. To elucidate the enzyme‐induced alterations in cellular dynamics and underlying apoptotic mechanism, intracellular H_2_O_2_ and ATP levels, as well as downstream expressions of HSP70, HSP90, and apoptosis‐related biomarkers, were measured. As shown in Figure 3G, intracellular ATP level in the Re@ReP‐G+L group was drastically downregulated and much lower than that in the Re@ReP‐G group, which was attributed to the hyperthermia‐enhanced enzymatic activity and glucose consumption. In contrast, there were no appreciable differences between the other treatment groups lacking GOx decoration and the control group in terms of ATP content. Meanwhile, slightly elevated levels of H_2_O_2_ were observed in cells treated with Re@ReP‐G (33.1%), with even higher levels observed in cells treated with Re@ReP‐G+L (46.8%) (Figure 3H). In contrast, the Re@ReP group (17.0%) showed apparent consumption of original H_2_O_2_ (reference control group, 27.4%), and the lowest H_2_O_2_ level was detected in the Re@ReP+L group (3.88%). These phenomenon was reconfirmed by corresponding CLSM results in Figure S14 (Supporting Information), which together supported the previous theory of enzyme‐accelerated degradation via the H_2_O_2_ actuator. Restricted by ATP deficiency, the expression of HSP70 and HSP90 (cellular defense) were reduced in Re@ReP‐G+L‐treated cells but markedly increased in Re@ReP+L‐treated cells to protect against hyperthermia injury, indicating that the delivered GOx had the ability to attenuate hyperthermia‐activated defense system in tumor cells (Figure 3I,J). The corresponding quantitative analysis in Figure S15 (Supporting Information) proved it again. Consequently, Re@ReP‐G+L treatment maximally activated pro‐apoptotic Bax while suppressing the expression of anti‐apoptotic Bcl‐2, leading to an increase in mitochondrial membrane permeability as demonstrated by the JC‐1 results, followed by Cyt‐C release (Figure 3K). These findings well verified the capacity of Re@ReP‐G plus laser to improve anticancer effect by depleting glucose and blocking the defense system in stimulating mitochondria‐regulated apoptosis.

### 2.4 In Vivo Pharmacokinetics and Biological Biodistribution

**Figure 4:**
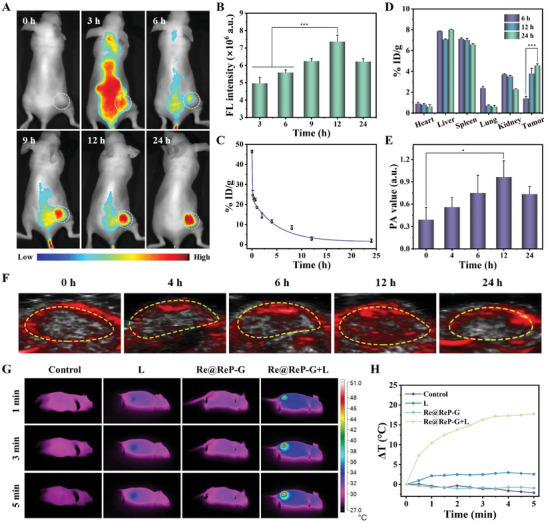
A) In vivo fluorescence imaging and B) corresponding fluorescence quantification of MCF‐7 tumor‐bearing mice after i.v. injection of Cy5‐labeled Re@ReP‐G. The white circles indicated the tumor region. C) Blood circulation and D) in vivo biodistribution of Re contents after i.v. injection of Re@ReP‐G at different time points. F) PA imaging and E) corresponding PA signal intensity of the tumors at different time points. G) Photothermal imaging and H) photothermal heating curves of MCF‐7 tumor‐bearing mice under different treatments. Statistical significance was calculated by one‐way ANOVA analysis. ^*^ *p* < 0.05; ^**^ *p* < 0.01; ^***^ *p* < 0.001.

Prior to investigating the in vivo therapeutic effects of Re@ReP‐G, their pharmacokinetics and biological distribution were evaluated on female BALB/c nude mice bearing MCF‐7 tumor or not. Re@ReP‐G showed a typical two‐compartment model with a half‐life of 3.42 h after a single intravenous (i.v.) injection via tail vein (**Figure** **4**C). As indicated in Figure 4A,B, mice treated with Cy5‐labeled Re@ReP‐G revealed a progressively increasing fluorescence signal at the tumor site that peaked at 12 h and still remained high at 24 h, which suggested that Re@ReP‐G was retained in the tumor for an extended period. In the end, major organs (heart, liver, spleen, lung, and kidney) and tumors were collected from the mice and immediately examined using an animal live imaging system. Figure S16 (Supporting Information) illustrated that Re@ReP‐G was mainly distributed in tumor tissue as opposed to other organs. In the meantime, an inductively coupled plasma emission spectrometer was used to assess changes in Re content in various tissue types over time. Re@ReP‐G efficiently accumulated in tumor tissue, with a high accumulation (3.79% ID g^−1^) observed at 12 h, which increased to 4.57% ID g^−1^ at 24 h (Figure 4D). On the other hand, elevated Re contents were detected to be excreted from the mice via the feces and urine pathways, suggesting that Re@ReP‐G could be degraded into small fragments or ions and then be eliminated from the body by the bile into the feces or the kidney to form urine (Figure S17, Supporting Information). Afterward, in vivo photoacoustic imaging (PA) of Re@ReP‐G at various timepoints was recorded, which revealed deep tissue penetration and fine spatial resolution. In agreement with the outcomes of in vivo fluorescence imaging, the PA signal intensity of tumor tissue continually rose from 4 to 12 h and then became less intense, with only a faint signal observed at 24 h (Figure 4E,F). Furthermore, infrared thermal imaging was also employed to monitor the real‐time temperature variations in tumor area during irradiation (Figure 4G,H). The temperature of tumor area rapidly increased by 17.8 °C when irradiated for 5 min in the Re@ReP‐G+L group, but by no more than 3 °C in the other groups under identical irradiation conditions, demonstrating the good in vivo photothermal conversion performance of Re@ReP‐G.

### 2.5 In Vivo Antitumor Assay

**Figure 5:**
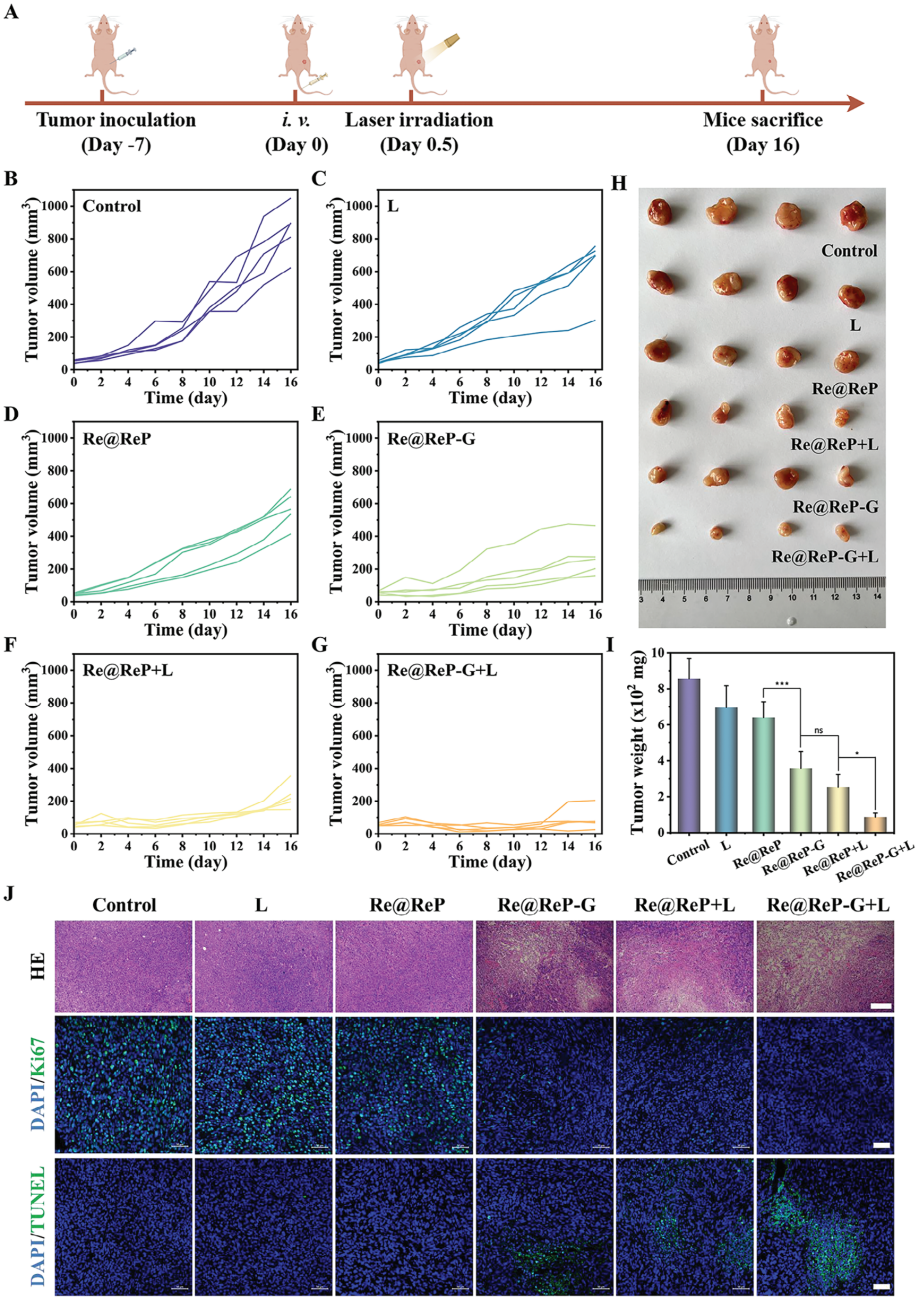
A) Demonstration of the treatment process. B–G) Individual tumor growth curves of mice in different groups including 1) Control, 2) L, 3) Re@ReP, 4) Re@ReP‐G, 5) Re@ReP+L, and 6) Re@ReP‐G+L. H) Digital images and I) weights of mice tumors after different treatments. J) HE staining (scale bar: 200 µm), Ki67 immunofluorescence staining (scale bar: 50 µm), and TUNEL staining (scale bar: 50 µm) for pathological changes and cellular proliferation in tumor tissues collected from different groups. Statistical significance was calculated by one‐way ANOVA analysis. ^*^ *p* < 0.05; ^**^ *p* < 0.01; ^***^ *p* < 0.001.

The in vivo anticancer efficacy of Re@ReP‐G under 1064 nm laser stimulation was investigated in MCF‐7 tumor‐bearing mice (*n =* 5) following the treatment procedure depicted in **Figure** **5**A. In particular, the laser (1 W cm^−2^) was executed for 5 min after 12 h of i.v. injection, and body weights and tumor volumes were tracked every two days for the next 16 days. The variations in relative tumor volume over time revealed that both the L and Re@ReP groups clearly exhibited rapidly expanding tumors, whereas the Re@ReP‐G and Re@ReP+L groups exhibited distinctly suppressed tumor growth, which suggested that single enzymatic or photothermal therapy was efficacious against tumors (Figure 5B–G; Figure S18A, Supporting Information). In contrast, the Re@ReP‐G+L group showed almost complete tumor regression owing to hyperthermia‐enhanced enzymatic activity, downregulated HSP, and amplified apoptosis. Real‐time photographic images of isolated tumors acquired at the end of the experiment and related weight data supported the aforementioned findings (Figure 5H,I). In addition, neither the treatment nor the control groups showed any noticeable changes in body weight during the therapy period (Figure S18B, Supporting Information). The strongest tumor‐killing and anti‐proliferation capabilities were verified for the Re@ReP‐G+L group by immunofluorescence staining of tumor slices with hematoxylin and eosin (HE), Ki67 antibody, and terminal deoxynucleotidyl transferase‐mediated dUTP nick end labeling (TUNEL) (Figure 5J).

### 2.6 In Vivo Biosafe Assay

**Figure 6:**
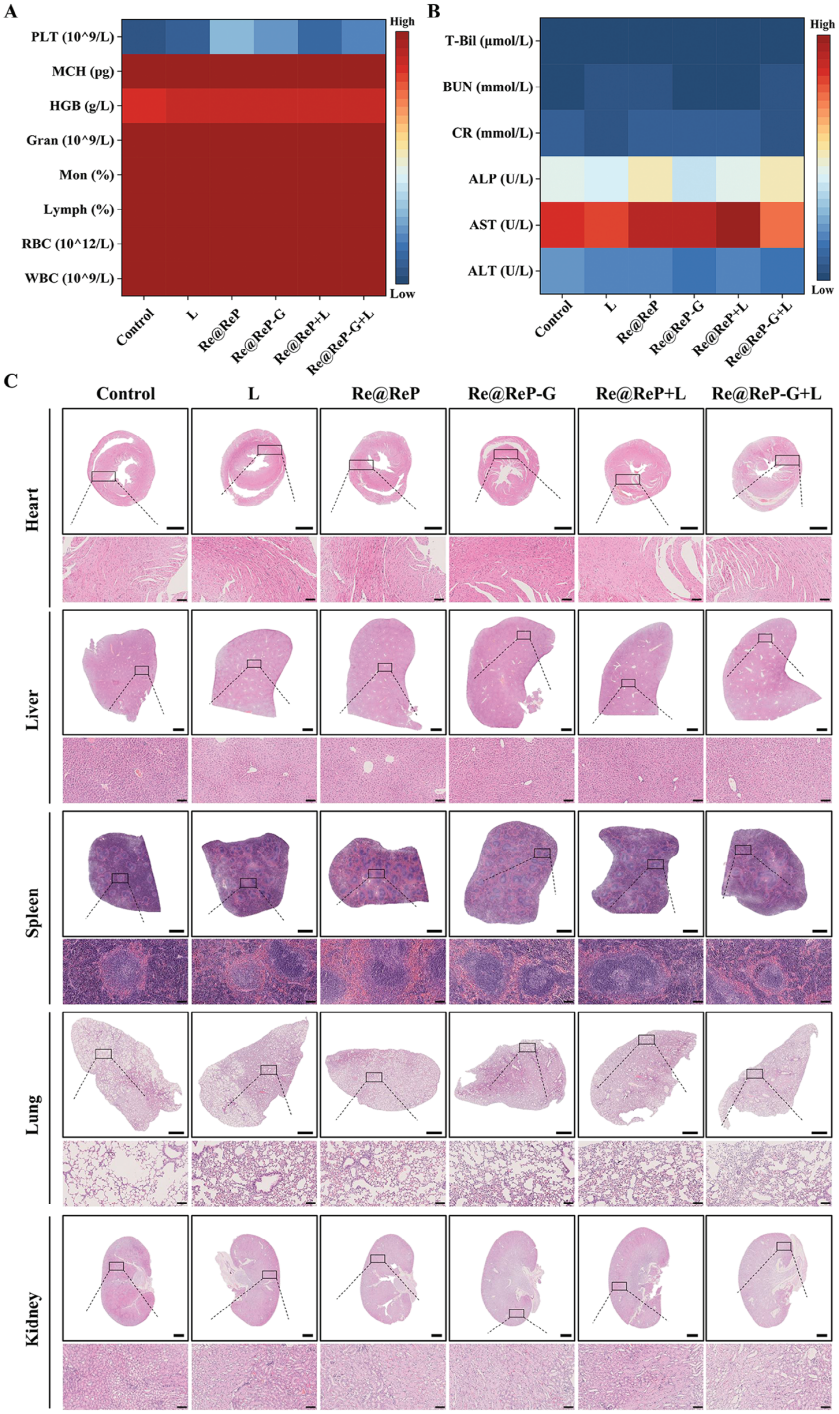
A) Hematology analysis and B) blood biochemistry of mice with different treatments. C) Histopathological examinations of major organs from mice with different treatments by HE staining, scale bar: 1000 µm. The scale bar for local magnification is 100 µm. Data represent means ± SD (n = 3). Statistical significance was calculated by one‐way ANOVA analysis. ^*^ *p* < 0.05; ^**^ *p* < 0.01; ^***^ *p* < 0.001.

The low hemolysis rate and normal complete blood count of Re@ReP‐G indicated the excellent hemocompatibility and biosafety (**Figure** **6**A; Figure S19, Supporting Information). None of the blood indicators for any of the treatment groups showed any observable differences when compared with the control group, suggesting the negligible renal and hepatic toxicities (Figure 6B). According to the results of the HE staining of the primary organs (heart, liver, spleen, lung, and kidney) of the mice sacrificed following in vivo anticancer therapy, no damages or inflammatory lesions were seen, which indicated that no evident side effects were associated with these treatments (Figure 6C).

## 3 Conclusion

In this study, we presented a novel Re@ReP‐G‐based “two birds with one stone” therapeutic strategy for hyperthermia‐enhanced enzyme‐mediated tumor apoptosis and enzyme‐promoted metabolic clearance. The delivered GOx catalyzed glucose and ATP depletion while improving H_2_O_2_ generation in the tumor microenvironment, which then attenuated the innate tumor defense system by downregulating HSP70 and HSP90 expression and increasing tumor susceptibility to apoptosis by reversing the anti‐apoptotic/pro‐apoptotic balance. The higher H_2_O_2_ concentration, on the other hand, stimulated rhenate formation and accelerated in vivo metabolism by degrading Re nanoclusters in Re@ReP‐G nanostructures from the outside in. More significantly, the addition of exogenous laser further boosted those enzymatic processes and degradation rates in vitro and in vivo, as well as the strongest anticancer efficacy and the fastest elimination in the Re@ReP‐G+L group. Collectedly, the Re@ReP‐G developed here not only provides an efficient approach for modulating tumor defense systems but also contributes to the clinical design of potential nanomedicines that simultaneously exhibit high therapeutic outcomes and low side effects.

## Conflict of Interest

The authors declare no conflict of interest.

## Supporting information

Supporting Information

## Data Availability

The data that support the findings of this study are available from the corresponding author upon reasonable request.
