# Supplementary material for: Enzyme‐Empowered “Two Birds with One Stone” Strategy for Amplifying Tumor Apoptosis and Metabolic Clearance
Source: Adv Sci (Weinh). 2024 Mar 6;11(18):2308251. doi: 10.1002/advs.202308251 (PMC11095162; doi:10.1002/advs.202308251)
Supplement: Supplementary file 1 — Supporting Information [file ADVS-11-2308251-s001.pdf]

ADVANCED SCIENCE
Open Access

Supporting Information

for *Adv. Sci.*, DOI 10.1002/advs.202308251

Enzyme-Empowered "Two Birds with One Stone" Strategy for Amplifying Tumor Apoptosis and Metabolic Clearance

Hanyue Li, Yihui Li, Lina Su, Ke Zheng, Yue Zhang, Jing Li, Feng Lv, Mengjie Huang, Ting Chen, Hanjie Zhang, Zhaoqing Shi, Dunwan Zhu, Xia Dong, Weiwei Zeng* and Lin Mei**

Supporting Information

Enzyme-Empowered “Two Birds with One Stone” Strategy for Amplifying Tumor Apoptosis and Metabolic Clearance

Hanyue Li, Yihui Li, Lina Su, Ke Zheng, Yue Zhang, Jing Li, Feng Lv, Mengjie Huang, Ting Chen, Hanjie Zhang, Zhaoqing Shi, Dunwan Zhu, Xia Dong, Weiwei Zeng*, Lin Mei**

Supporting Information for

Enzyme-Empowered "Two Birds with One Stone" Strategy for Amplifying Tumor Apoptosis and Metabolic Clearance

Hanyue Li, Yihui Li, Lina Su, Ke Zheng, Yue Zhang, Jing Li, Feng Lv, Mengjie Huang, Ting Chen, Hanjie Zhang, Zhaoqing Shi, Dunwan Zhu, Xia Dong, Weiwei Zeng*, Lin Mei**

H. Li, F. Lv, Y. Zhang, J. Li, T. Chen, H. Zhang, Z. Shi, D. Zhu, X. Dong, W. Zeng, L. Mei
State Key Laboratory of Advanced Medical Materials and Devices, Tianjin Key Laboratory of Biomedical Materials, Institute of Biomedical Engineering, Chinese Academy of Medical Sciences and Peking Union Medical College, Tianjin 300192, PR China
E-mail: dongxia@bme.pumc.edu.cn; zengww7@mail2.sysu.edu.cn; meilin@bme.pumc.edu.cn

Y. Li
Guangdong Provincial Key Laboratory of Malignant Tumor Epigenetics and Gene Regulation, Guangdong-Hong Kong Joint Laboratory for RNA Medicine, Sun Yat-Sen Memorial Hospital, Sun Yat-Sen University, Guangzhou 510120, PR China

L. Su, L. Mei
Department of Pharmacy, Qujing Medical College, Qujing 655000, PR China

K. Zheng
School of Materials Science and Engineering, Dongguan University of Technology, Dongguan 523808, PR China

M. Huang
Department of Nephrology, First Medical Center of Chinese PLA General Hospital, National Key Laboratory of Kidney Diseases, National Clinical Research Center for Kidney Diseases, Beijing Key Laboratory of Kidney Diseases Research, Beijing 100853, PR China

Experimental Section

***Materials*:** Poly(vinyl alcohol) (PVA), pyrrole (99%), pyrrole-3-carboxylic acid (95%), Sulfo-cyanine5 N-hydroxysuccinimide ester, sodium borohydride (NaBH4, 98%), N-Hydroxysuccinimide (NHS, 98%), and N-(3-Dimethylaminopropyl)-N′-ethylcarbodiimide hydrochloride (EDC, 98%) were purchased from Aladdin. Rhenium heptoxide (Re_2O_7, 99.99%) and glucose oxidase (GOx) were obtained from Macklin. BES-H_2O_2-Ac was acquired from FUJIFILM Wako Pure Chemical Corporation Sigma-Aldrich. Anti-HSP70 Monoclonal Antibody, Anti-HSP90 β Monoclonal Antibody, 2-(4-Amidinophenyl)-6-indolecarbamidine dihydrochloride (DAPI) was purchased from Solarbio. Goat Anti-Rabbit IgG(H+L) Alexa Fluor 488 was obtained from Shanghai share-bio Biotechnology. Bcl-2 Rabbit mAb, Bax Rabbit mAb, and Cytochrome c Rabbit mAb were purchased from Cell Signaling. Hydrogen peroxide assay kit, cell counting kit-8 (CCK-8), annexin V-FITC apoptosis detection kit, mitochondrial membrane potential assay kit, HRP-labeled Goat Anti-Rabbit IgG (H+L), and enhanced ATP assay kit were obtained from Beyotime Biotechnology.

***Synthesis of ReP and Re@ReP*:** In a 100 round-bottom flask, 50 mg PVA and 19 mL deionized (Di) water were added, and the temperature was increased to 90 °C with gentle stirring for 1 h. Then 100 mg Re_2O_7 pre-dissolved in 1 mL ethanol was slowly added and continuously stirred at 90 °C. After 0.5 h, 100 μL pyrrole monomer and 80 mg pyrrole-3-carboxylic acid dissolved in 1 mL Di water were added to the above mixture and stirred for 23 h. For the preparation of rhenic acid-doped polypyrrole (ReP), the mixture was cooled to room temperature and then centrifuged at 18000 rpm for 10 min to collect the precipitate, which was washed three times with Di water. For the preparation of rhenium nanoclusters-doped polypyrrole (Re@ReP), the mixture was cooled to room temperature and then 4 mL $NaBH_4$ (4 mg mL^{-1}) was added and stirred for 3 h. The final product was centrifuged at 18000 rpm for 10 min to collect the precipitate, which was washed three times with Di water.

***Synthesis of Re@ReP-G*:** At first, 10 mg Re@ReP was completely dispersed in 8 mL Di water, and then 2 mL of a mixed aqueous solution of EDC/NHS (EDC: 10 mg; NHS: 6.5 mg) was added and stirred for 1 h. Subsequently, 5 mg GOx (pre-dissolved in 1 mL Di water) was added dropwise to the above mixture and stirred overnight at room temperature. Finally, the mixture was purified in a dialysis bag (300 kD) for 48 h. The obtained Re@ReP-G was stored at 4 °C for further experiments.

Characterization**:** Transmission electron microscopy (TEM) images were acquired on a JEM-2100UHR microscope (JEOL, Japan). X-ray photoelectron spectroscopy (XPS, ESCALAB 250Xi, Japan), energy-dispersive X-ray spectroscope (EDS, Inca X-MAX, Oxford, UK), and Fourier transform infrared spectrophotometer spectrum (FTIR, Nexus 470, Nicolet, Madison, WI, USA) were applied to analyze the chemical constitutions of different formulations. X-ray diffraction (XRD, Bruker D8, Germany) was performed to analyze the crystal structure of different formulations. UV-vis-NIR spectrum was investigated by using an UV/vis spectrometer (Lambda 35, PerkinElmer, Singapore). The hydrodynamic particle sizes and zeta potentials of different formulations were monitored by dynamic light scattering (Zen 3600 Zetasizer, Malvern, England).

Enzymatic activity of Re@ReP-G**:** 225 μg/mL of Re@ReP-G was stirred in PBS solution with or without glucose (10 mM) at various temperature (25 °C and 45 °C). At different time points (0, 10, 20, 30, 40, 50, 60, 75, 90, 105, 120, 150, and 180 min), the absorbance of the supernatant at 560 nm was measured using a hydrogen peroxide detection kit to calculate the concentration of H_2O_2. The corresponding pH values were measured using a pH meter.

Photothermal performance of Re@ReP-G**:** Different concentrations of Re@ReP-G aqueous solutions (0, 25, 50, 100, and 200 μg mL^{-1}) were exposed to an 808 nm or 1064 nm laser at various power densities (0.5, 1.0, 1.5, and 2.0 W cm^{-2}) for 5 min. An IR thermal camera (Ti480U, Fluke, USA) was used to monitor the temperature change of solutions in real time. Furthermore, the photothermal stability and heating-cooling curve of Re@ReP-G aqueous solution (100 μg mL^{-1}) were assessed under irradiation of an 808 nm or 1064 nm laser (1.0 W cm^{-2}) for five on/off cycles (on: 6 min, off: 9 min).

In vitro degradation experiments**:** 100 μg mL^{-1} of Re@ReP-G was first dispersed in pure PBS, PBS with H_2O_2 (100 mM), or PBS with glucose (10 mM) and then co-cultured at 37 °C for various times. The laser irradiated group was irradiated with a 1064 nm laser with a power density of 1 W cm^{-2} for 5 min after 1 h of co-incubation. At defined time points (6, 12, and 24 h), certain mixtures were extracted and centrifuged for TEM observation. In the end, XPS analysis was performed to identify changes in composition.

Cellular uptake**:** To assess the cellular internalization behavior of Re@ReP-G, the nanoparticles were labeled with the near-infrared dye Cy5 and co-incubated with MCF-7 cells

for different times (0, 2, 4, 8, and 12 h) at a Cy5 concentration of 5 μg mL^{-1}. For microscopy imaging, the nuclei were stained with DAPI and imaged by confocal laser scanning microscopy (CLSM, LSM710, Carl ZEISS, Germany). For quantitative analysis, the cells were collected and analyzed by flow cytometry (Celesta, BD, USA).

***Cytotoxicity assay*:** The cytotoxicity of Re@ReP and Re@ReP-G was conducted by the standard Cell Counting Kit-8 (CCK-8) assay. Briefly, cancer cells MCF-7 or normal cells 3T3 were seeded in 96-well plates at a density of 5 x 10^3 cells per well and co-cultured for 24 h at 37 °C with 5% CO_2. Subsequently, the medium was replaced with fresh medium containing different concentrations Re@ReP (0, 12.5, 25, 50, 100, 200, and 400 μg mL^{-1}) or Re@ReP-G (0, 12.5, 25, 50, 100, and 200 μg mL^{-1}). After 24 or 48 h of co-incubation, cells were washed twice with PBS and analyzed using the standard protocol of CCK-8 assay.

To further evaluate in vitro photothermal cytotoxicity of Re@ReP-G, MCF-7 cells were treated with PBS, Re@ReP, or Re@ReP-G (equal Re@ReP concentration: 50 or 75 μg mL^{-1}) for 4 h. Afterward, cells in the laser-required groups were exposed to a 1064 nm laser with a power density of 1 W cm^{-2} for 5 min and continued to be incubated for 24 h. Finally, the cells were washed twice with PBS and cell viability was measured according to the standard protocol of CCK-8 assay. For cell apoptosis assays, MCF-7 cells were treated as described above and then incubated with Annexin V-FITC and propidium iodide (PI) in the dark according to the manufacturer's instructions. Finally, the cells were washed with PBS and immediately analyzed using flow cytometer (Celesta, BD, USA).

***Intracellular ATP and H_2O_2*:** Cellular ATP levels were detected using the enhanced ATP assay kit. In brief, MCF-7 cells were treated with PBS, Re@ReP, or Re@ReP-G (equal Re@ReP concentration: 75 μg mL^{-1}) for 24 h. Next, the cells were washed, lysed, and centrifuged to collect the suspension, which was analyzed using the ATP assay kit according to the manufacturer's instructions. As for the assessment of cellular H_2O_2 levels, MCF-7 cells were treated as described above and then incubated with DMEM containing BES-H_2O_2-Ac (10 μM) for 30 min. Finally, the cells were analyzed using flow cytometer and visualized by CLSM.

***Intracellular HSP and apoptosis index*:** MCF-7 cells were seeded in confocal dish (1.5 × 10^5 per well) or 6-well plates (3 × 10^5 per well) and cultured for 24 h. Then, the media were replaced with fresh DMEM medium containing 75 μg mL^{-1} of Re@ReP or Re@ReP-G. After 4 h of incubation, cells in the groups requiring laser irradiation were exposed to a 1064 nm laser (1 W

cm^{-2}, 5 min) and continued to be incubated for 2 h. For CLSM observation, the cells on confocal dish were co-incubated with Anti-HSP70 Monoclonal Antibody or Anti-HSP90 β Monoclonal Antibody for 2 h, and then co-incubated with Goat Anti-Rabbit IgG(H+L) Alexa Fluor 488 for 1 h. After that, the cells were washed and visualized by CLSM. As for western blotting assay, the cells on 6-well plates were collected and lysed in an ice-bath, then the total proteins were extracted with lysate buffer and quantified with different protein assay kits.

***Animal model*:** All animal experiments were approved by the Institutional Animal Care and Use Committee of Chinese Academy of Medical Sciences-Peking Union Medical College Institute of Radiation Medicine (Approval number: IRM-DWLL-2023203). Female BALB/c nude mice (17-20 g, 4-6 weeks old) were obtained from SPF Biotechnology Co. Ltd. All animal experiments were rigorously conducted in accordance with the regulations on animal use and care. 5×10^6 MCF-7 cells suspended in PBS solution were subcutaneously injected into the right back of BALB/c nude mice. When the tumor became distinct and the tumor volume reached ~80 mm^3, the nude mice were randomly divided into different groups.

***In vivo biological distribution*:** Nude mice bearing MCF-7 tumor were intravenously injected with Cy5-labelled Re@ReP-G at Cy5 dose of 1.5 mg kg^{-1}. At defined time intervals (3, 6, 9, 12, and 24 h) after injection, nude mice were imaged by animal live imaging system (CRi, USA). At the end of the experiment, all nude mice were sacrificed to collect tumors and major organs (heart, liver, spleen, lung, and kidney) for ex vivo imaging.

***In vivo photoacoustic imaging*:** For in vivo photoacoustic (PA) imaging, nude mice bearing MCF-7 tumor were administrated with Re@ReP-G at a dose of 12.5 mg kg-1 via intravenous injection. At different time points (0, 4, 6, 12, and 24 h), PA images of the tumor site and quantitative analysis of PA signal intensities were performed and the signs were recorded.

***In vivo antitumor therapy*:** To investigate the therapeutic efficacy in vivo, MCF-7 tumor-bearing nude mice were randomly divided into six groups (n=5): (1) PBS group (Control), (2) Laser group (L), (3) Re@ReP group, (4) Re@Re-G group, (5) Re@ReP+Laser group (Re@ReP+L), (6) Re@ReP-G+Laser group (Re@ReP-G+L). In groups (1) and (2), nude mice were injected intravenously with PBS, a 12.5 mg kg^{-1} dose of Re@ReP in groups (3) and (5), and the same dose of Re@ReP as Re@ReP was injected intravenously with Re@ReP-G in groups (4) and (6). For the groups that needed laser treatment, a 1064 nm laser (1 W cm^{-2}, 5

min) were irradiated to the tumor sites 12 h after injection. Furthermore, each mouse's body weight and tumor volume were assessed every two days for 16 days, and the tumor volume was determined using the formula tumor volume =1/2 × (tumor width)^2 × (tumor length). At 16 days, all tumors and organs (heart, liver, spleen, lung, and kidney) were extracted for further histological analysis.

***In vivo pharmacokinetics*:** The nude mice bearing MCF-7 tumor were intravenously injected with Re@ReP-G (12.5 mg kg^{-1}) (*n=3*). At specific time intervals (10, 20, 40, 60, 120, 240, 480, 720, and 1440 min) after injection, the blood of nude mice was collected for ICP to measure the Re concentration. The nude mice bearing MCF-7 tumor were intravenously injected with Re@ReP-G (12.5 mg kg^{-1}) (*n=3*). At specific time intervals (6, 12, and 24 h) after injection, the tumors and organs (heart, liver, spleen, lung, and kidney) of nude mice were collected for ICP to measure the Re concentration.

***Statistical analysis*:** All statistical analyses were used GraphPad Prism. The results of statistical analysis were presented as mean ± SD. Statistical significance was calculated by one-way ANOVA analysis. The statistical significance was defined as *p < 0.05; **p < 0.01; ***p < 0.001.

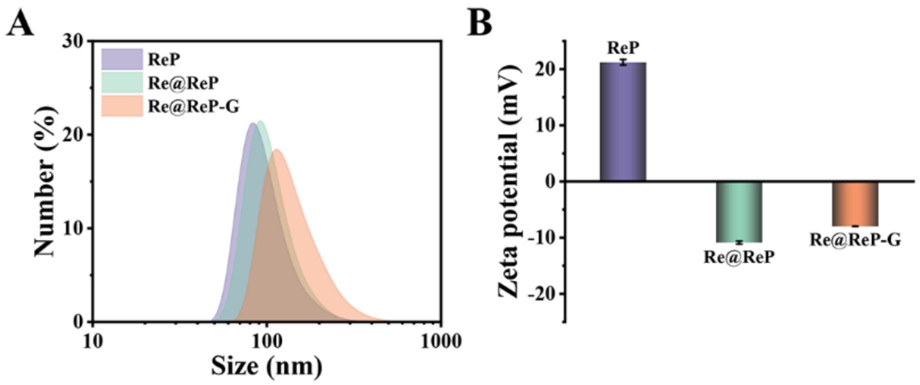

Figure S1. (A) Hydrodynamic particle size and zeta potentials of ReP, Re@ReP, and Re@ReP-G.

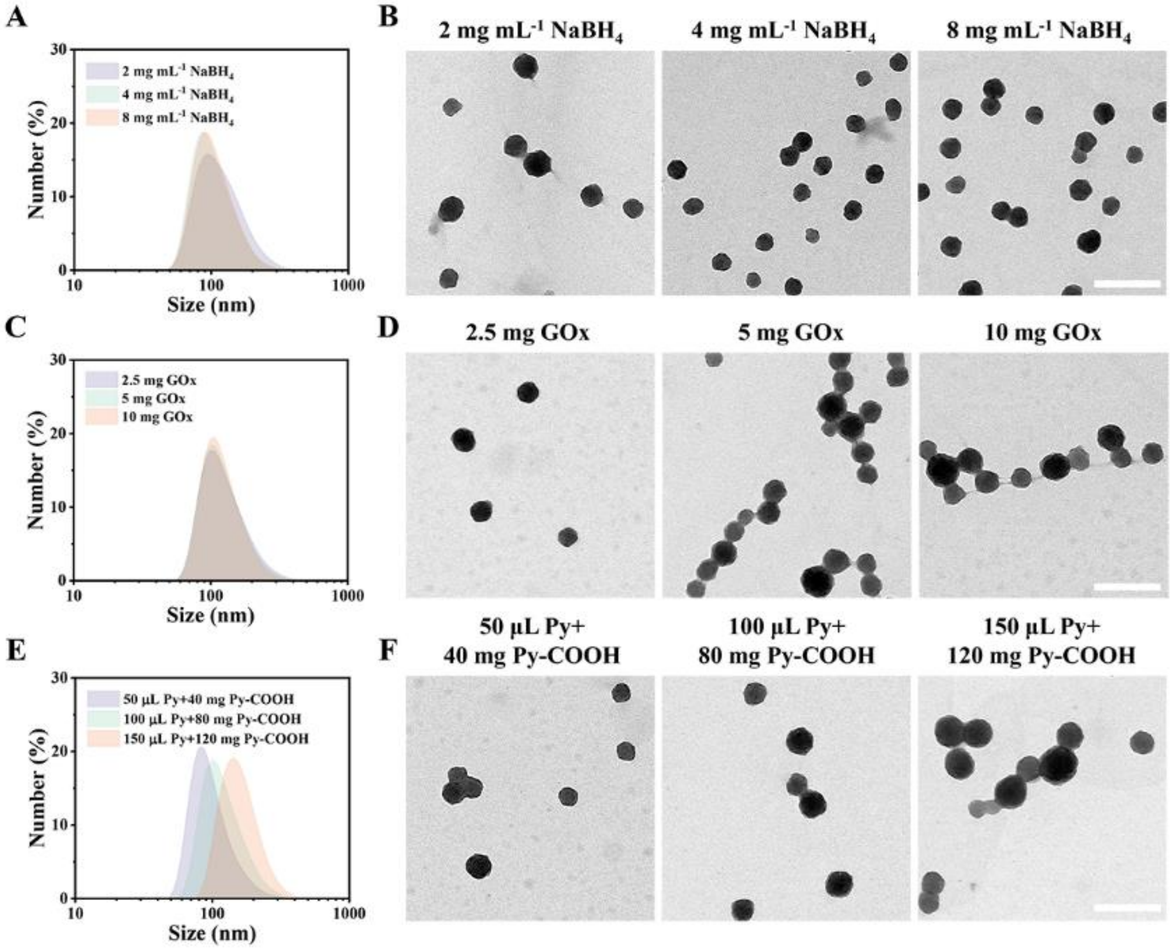

Figure S2. (A) Hydrodynamic particle size and (B) corresponding TEM images of Re@ReP with different adding amounts of $NaBH_4$. Scale bar: 200 nm. (C) Hydrodynamic particle size and (D) corresponding TEM images of Re@ReP-G with different adding amounts of GOx. Scale bar: 200 nm. (E) Hydrodynamic particle size and (F) corresponding TEM images of Re@ReP with different adding amounts of pyrrole (Py) and pyrrole-3-carboxylic acid (Py-COOH). Scale bar: 200 nm.

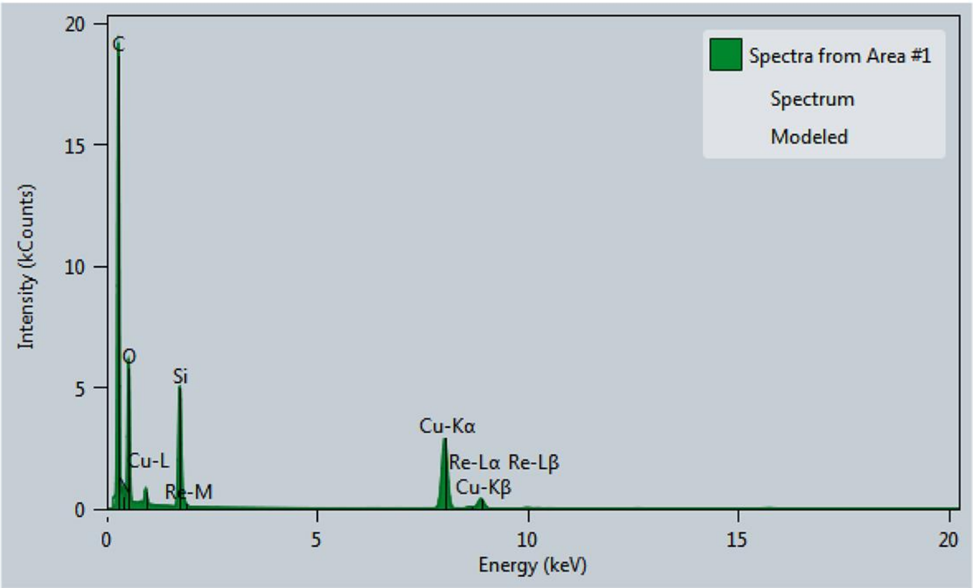

Figure S3. X-ray energy dispersive spectroscopy (EDS) of Re@ReP-G.

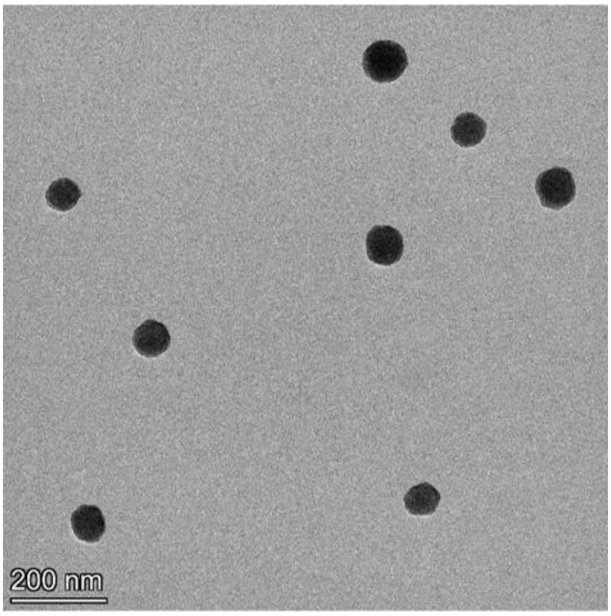

Figure S4. TEM image of Re@ReP-G.

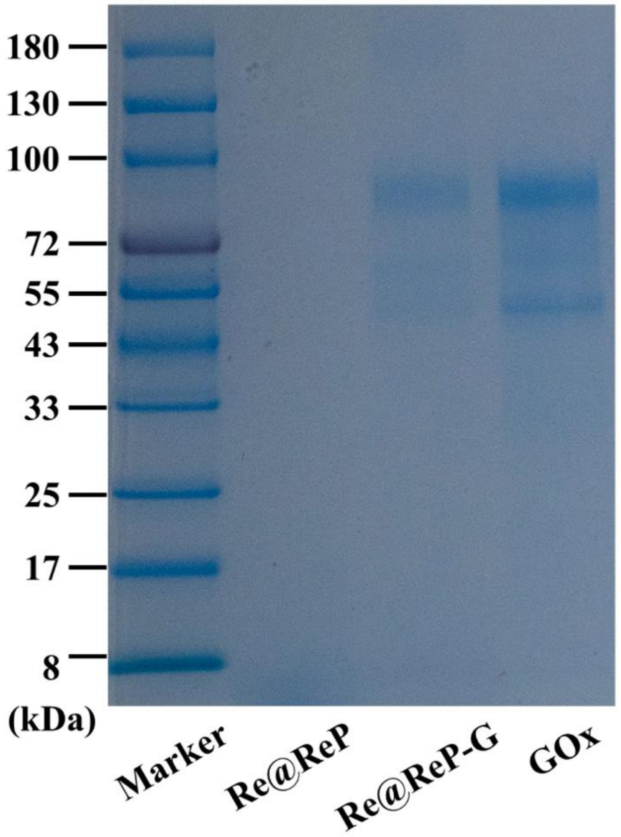

Figure S5. SDS-PAGE protein analysis of GOx, Re@ReP, and Re@ReP-G.

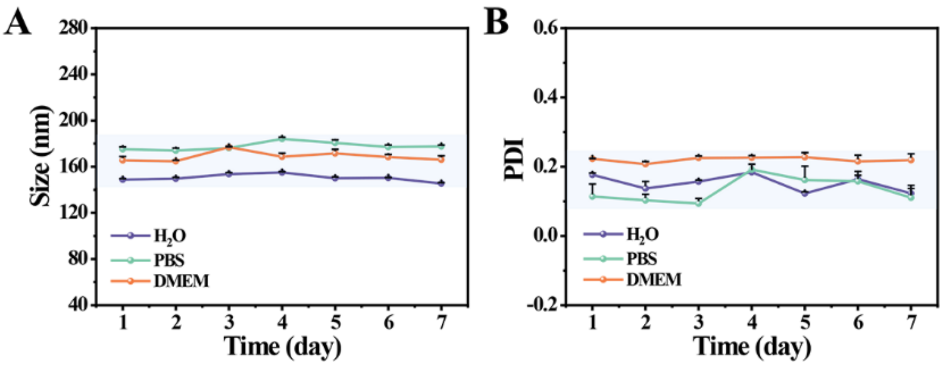

Figure S6. (A) Hydrodynamic diameter changes and (B) corresponding PDI changes of Re@ReP-G dispersed in different physiological media at varied time points.

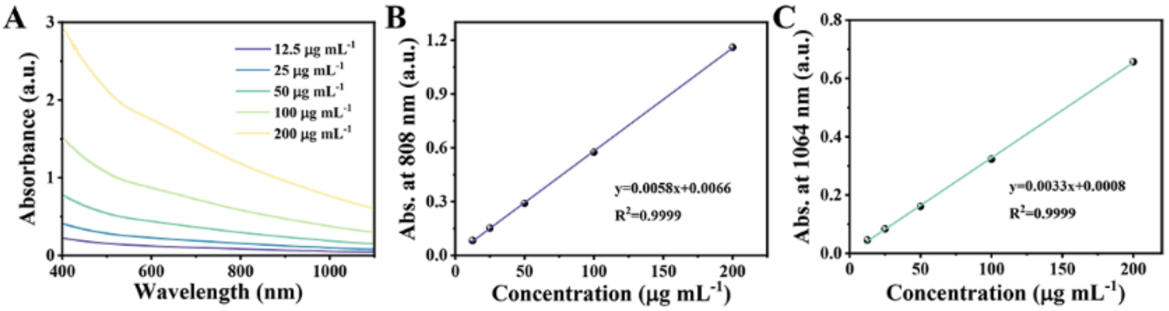

Figure S7. (A) UV-vis-NIR spectra of various concentrations of Re@ReP-G. Linear relationship between concentration and absorbance of Re@ReP-G at (B) 808 and (C) 1064 nm.

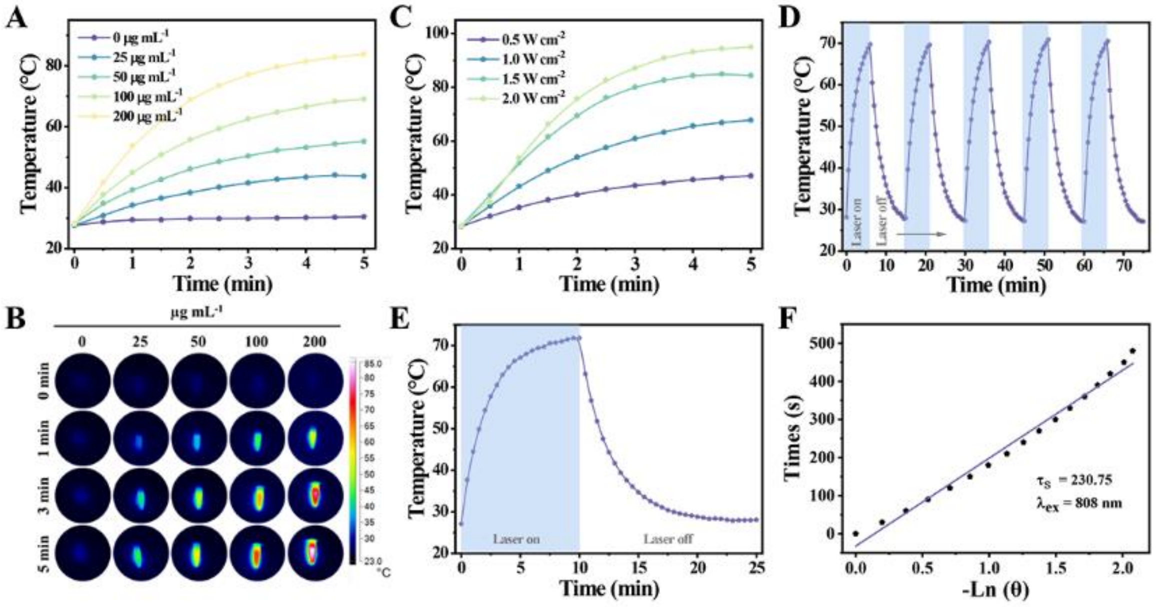

Figure S8. (A) Temperature profiles of different concentrations of Re@ReP-G under laser irradiation (808 nm, 1.0 W cm^{-2}, 5 min) and (B) the corresponding photothermal pictures. (C) Temperature profiles of Re@ReP-G exposed to different power densities (0.5-2.0 W cm^{-2}) of 808 nm laser. (D) Photothermal conversion stability of Re@ReP-G aqueous solution for five laser on/off cycles under the irradiation of 808 nm laser. (E) Photothermal heating and cooling curves of Re@ReP-G under 808 nm laser irradiation and (F) corresponding linear relationship between time and -lnθ from the cooling period.

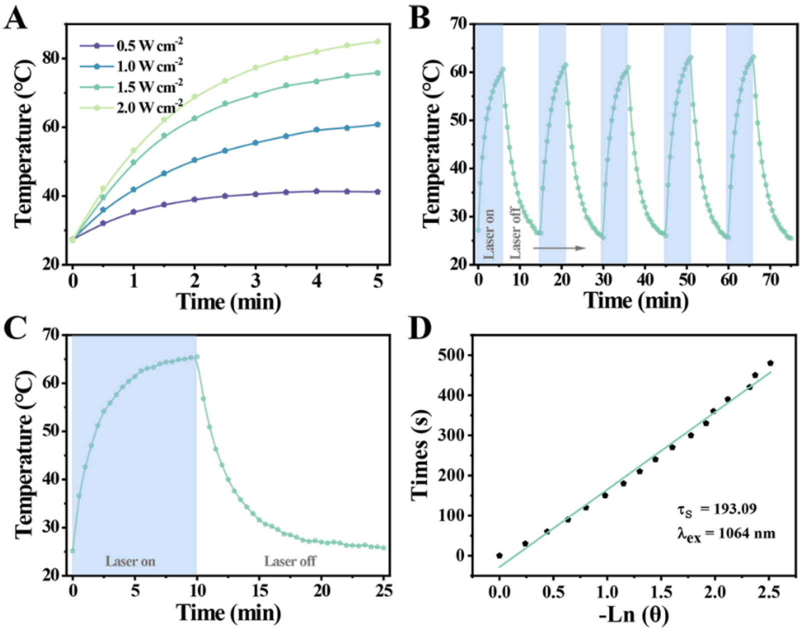

Figure S9. (A) Temperature profiles of Re@ReP-G exposed to different power densities (0.5-2.0 W cm^{-2}) of 1064 nm laser. (B) Photothermal conversion stability of Re@ReP-G aqueous solution for five laser on/off cycles under the irradiation of 1064 nm laser. (C) Photothermal heating and cooling curves of Re@ReP-G under 1064 nm laser irradiation and (D) corresponding linear relationship between time and -lnθ from the cooling period.

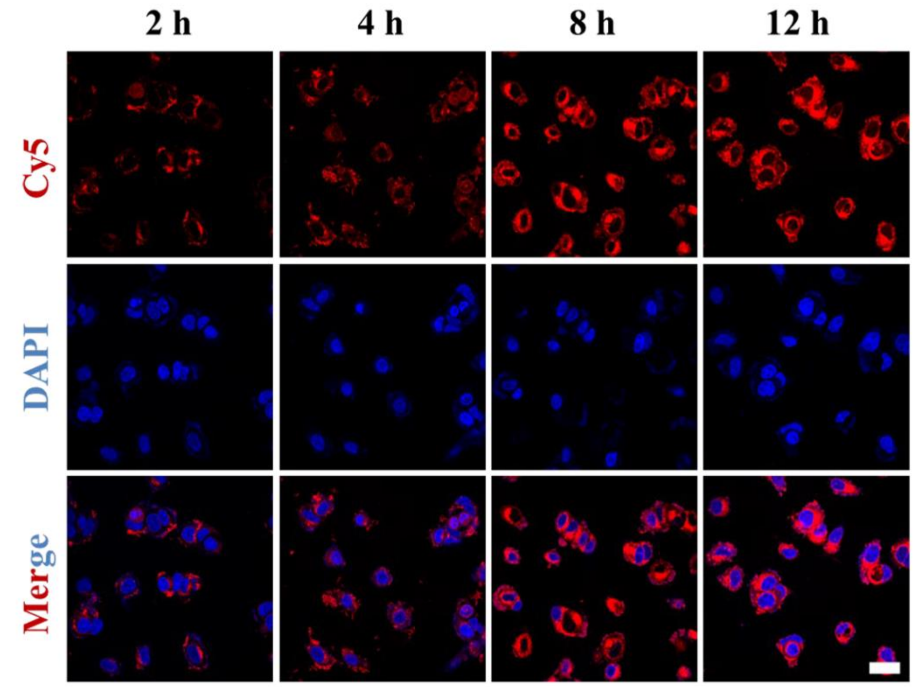

Figure S10. CLSM images of MCF-7 cells incubated with Cy5-labelled Re@ReP-G for different times (2, 4, 8, and 12 h), scale bar: 20 μm.

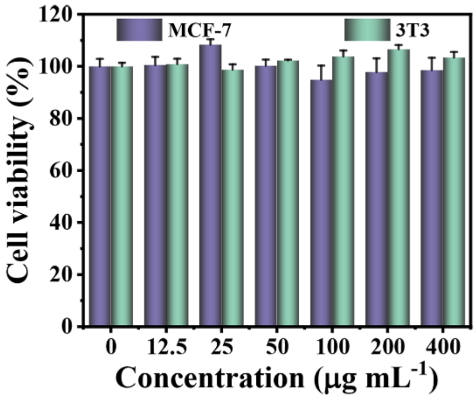

Figure S11. Cell viability of MCF-7 or 3T3 cells incubated with various concentrations of Re@ReP for 48 h.

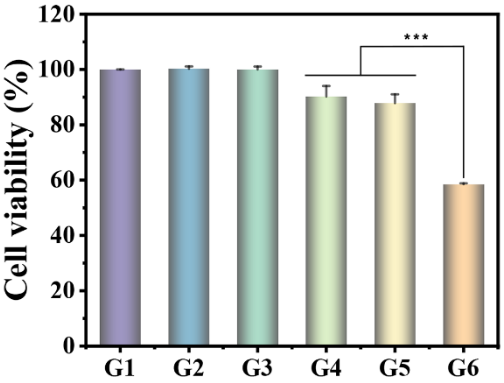

Figure S12. Cell viability of MCF-7 cells after different treatments at equal Re@ReP concentration of 50 μg mL^{-1}. Statistical significance was calculated by one-way ANOVA analysis. *P < 0.05; **P < 0.01; ***P < 0.001.

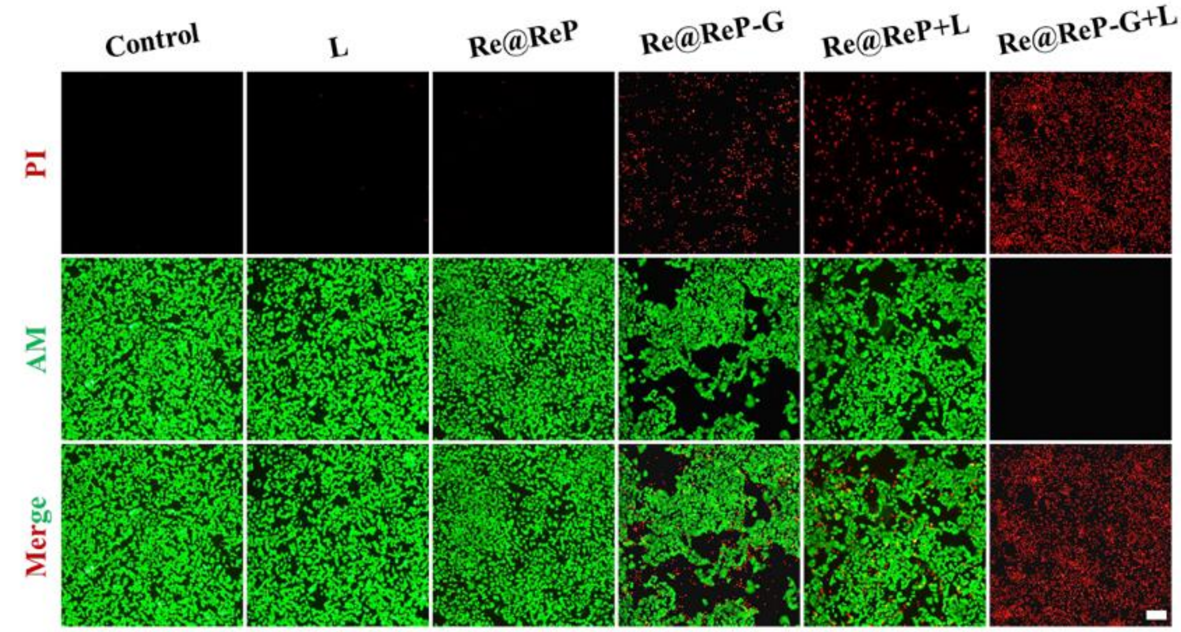

Figure S13. PI and calcein AM staining to identify live/dead cells (red: dead, green: live) with different treatments, scale bar: 100 μm.

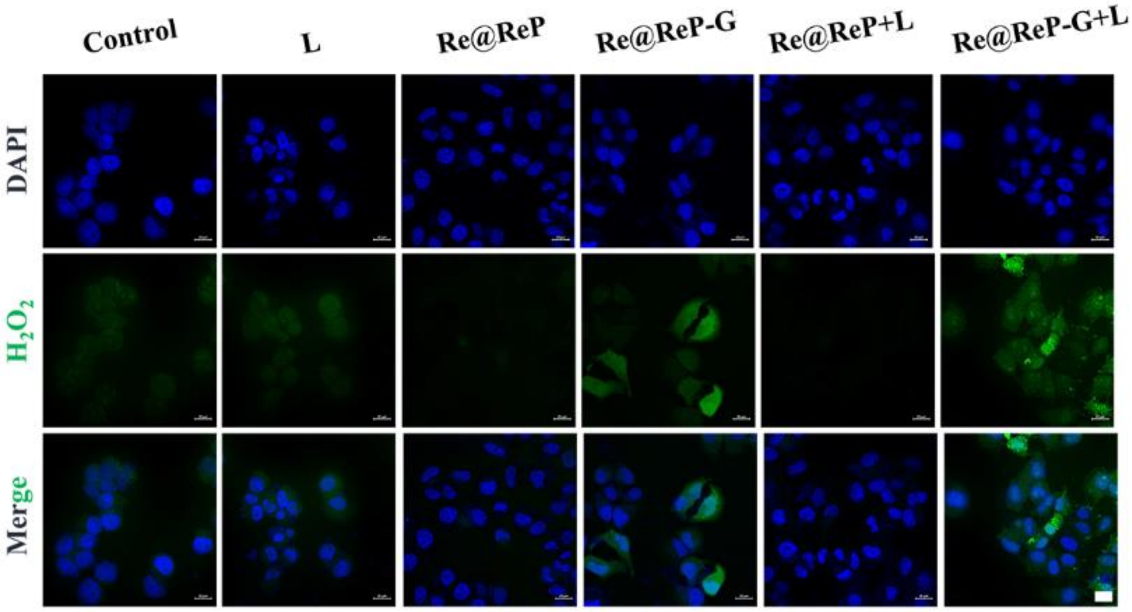

Figure S14. CLSM images of H_2O_2 levels in MCF-7 cells treated with different formulations, scale bar: 20 μm.

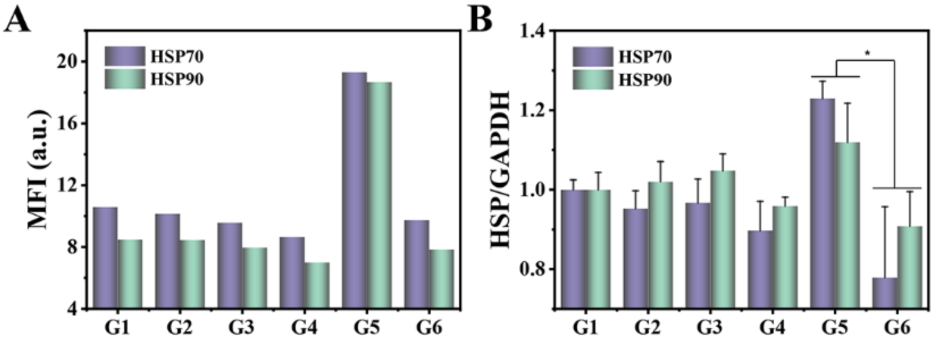

Figure S15. (A) Quantitative analysis of intracellular HSP70 and HSP90 fluorescence signals after different treatments. (B) Quantitative analysis of intracellular HSP70 and HSP90 protein expression after different treatments. G1: Control; G2: L; G3: Re@ReP; G4: Re@ReP-G; G5: Re@ReP+L; G6: Re@ReP-G+L. Statistical significance was calculated by one-way ANOVA analysis. *P < 0.05; **P < 0.01; ***P < 0.001.

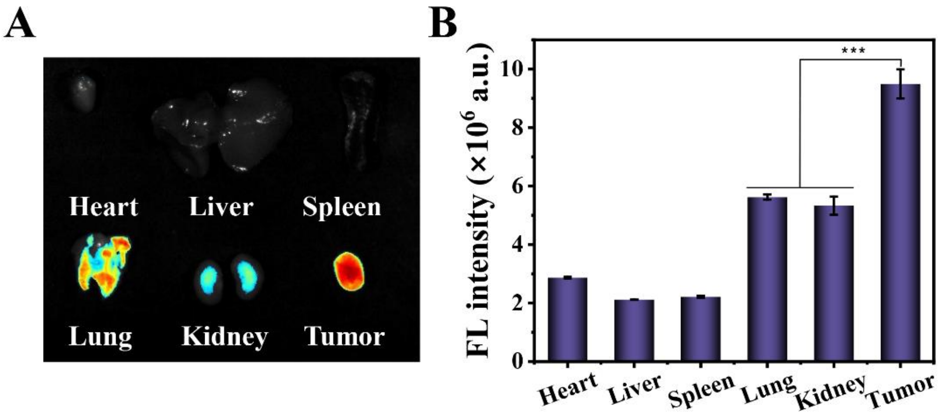

Figure S16. (A) Ex vivo fluorescence imaging and (B) corresponding fluorescence quantification of the tumor and major organs 24 h after injection of Cy5-labelled Re@ReP-G. Statistical significance was calculated by one-way ANOVA analysis. *P < 0.05; **P < 0.01; ***P < 0.001.

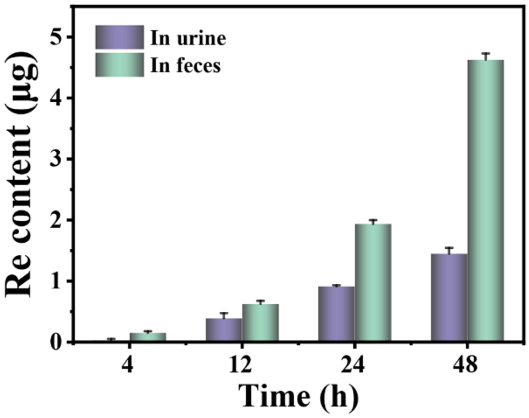

Figure S17. Detection of Re in urine and feces after injection of Re@ReP-G at various time intervals.

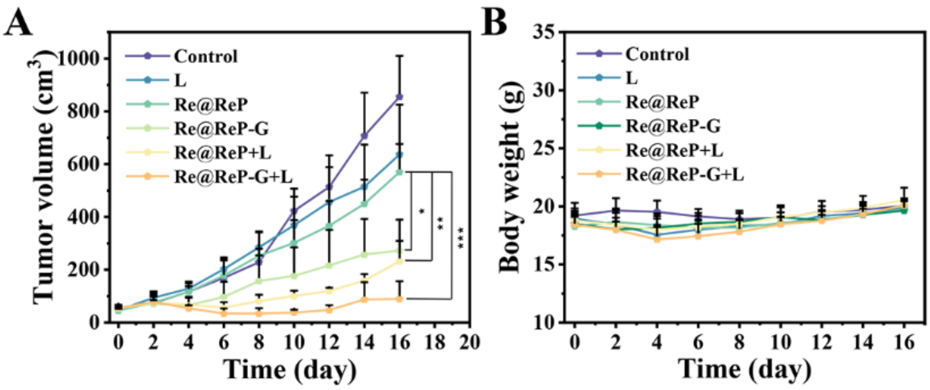

Figure S18. (A) Relative tumor volumes of mice in different groups, including 1) Control, 2) L, 3) Re@ReP, 4) Re@ReP-G, 5) Re@ReP+L, and 6) Re@ReP-G+L (*n* = 5, mean ± SD). (B) Body weight curves of mice with different treatments. Statistical significance was calculated by one-way ANOVA analysis. *$P < 0.05$; **$P < 0.01$; ***$P < 0.001$.

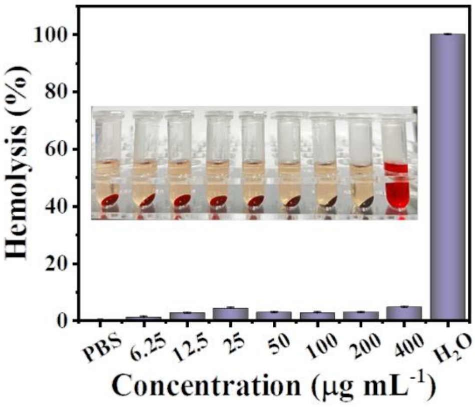

Figure S19. Hemolysis assay of red blood cells treated with water, PBS and Re@ReP-G dispersed in PBS at different concentrations.
